# Supplementary material for: Description of Trends over the Week in Alcohol-Related Ambulance Attendance Data
Source: Int J Environ Res Public Health. 2023 Apr 19;20(8):5583. doi: 10.3390/ijerph20085583 (PMC10138978; doi:10.3390/ijerph20085583)
Supplement: Supplementary file 1 [file ijerph-20-05583-s001.zip › Ambo_HAH_Figures S1-S8.docx]

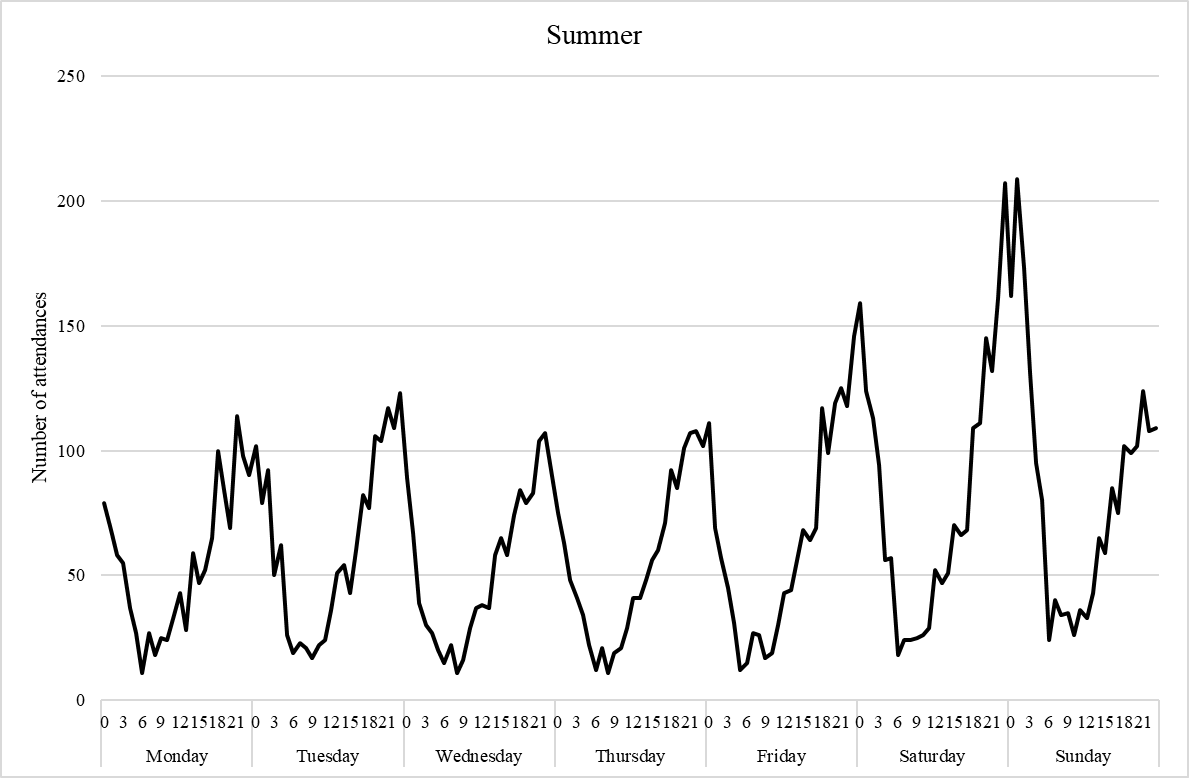

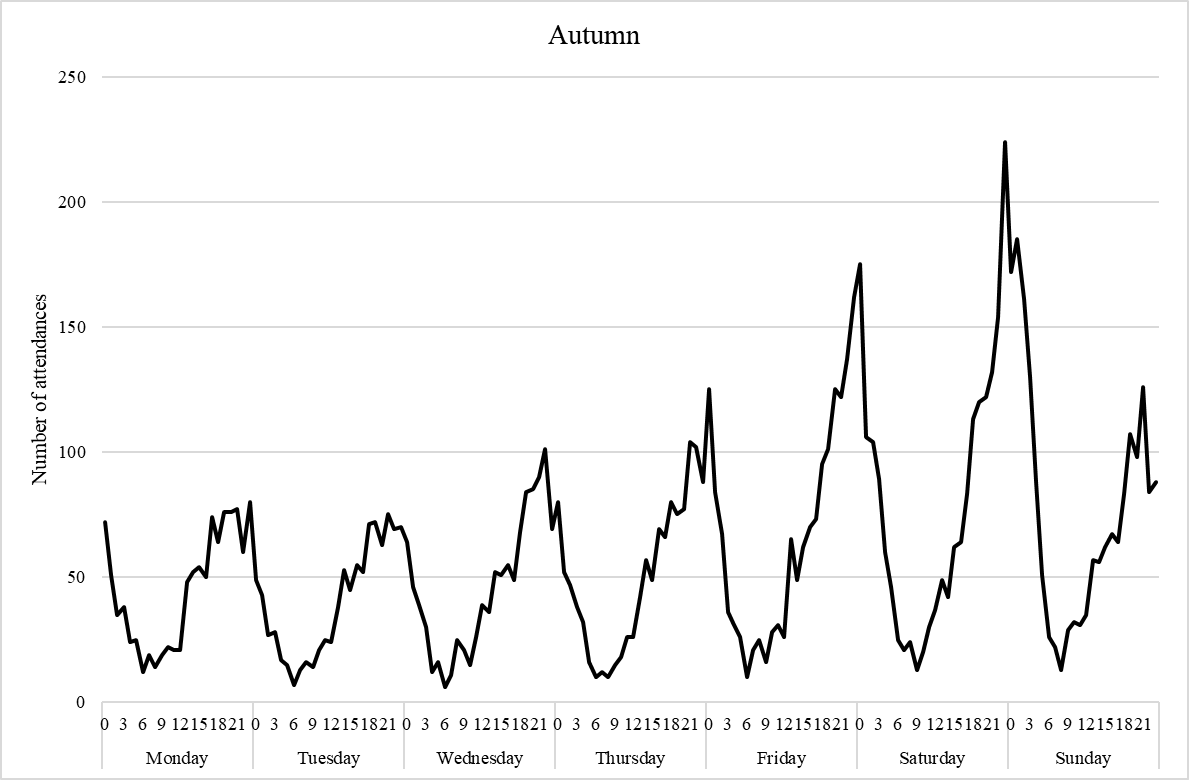


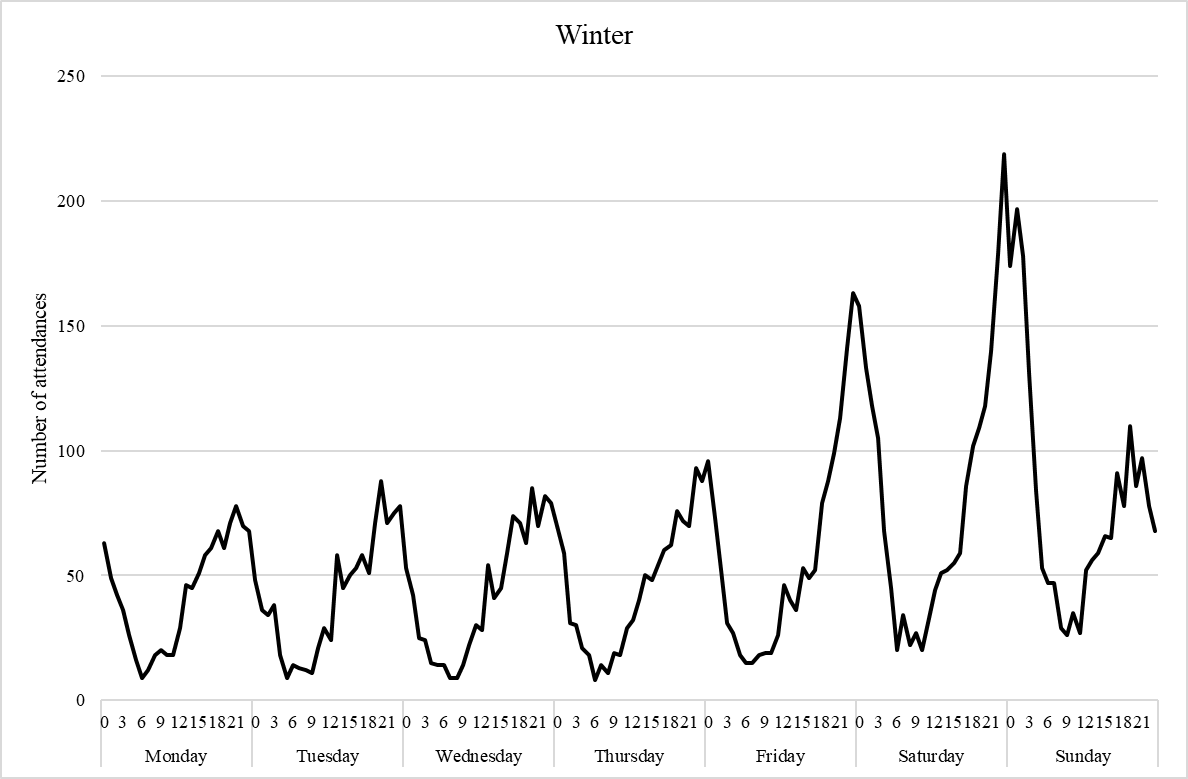

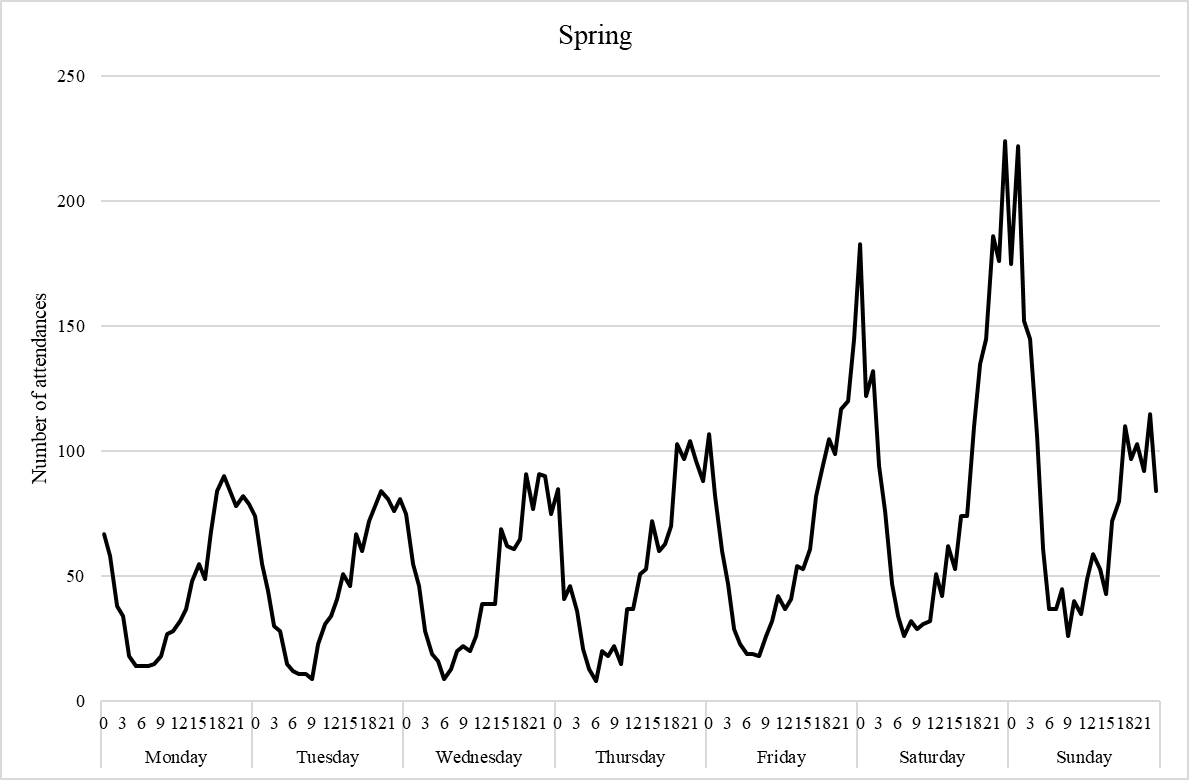


Figure S1. 2019 alcohol-involved ambulance attendances by season, Victoria, Australia


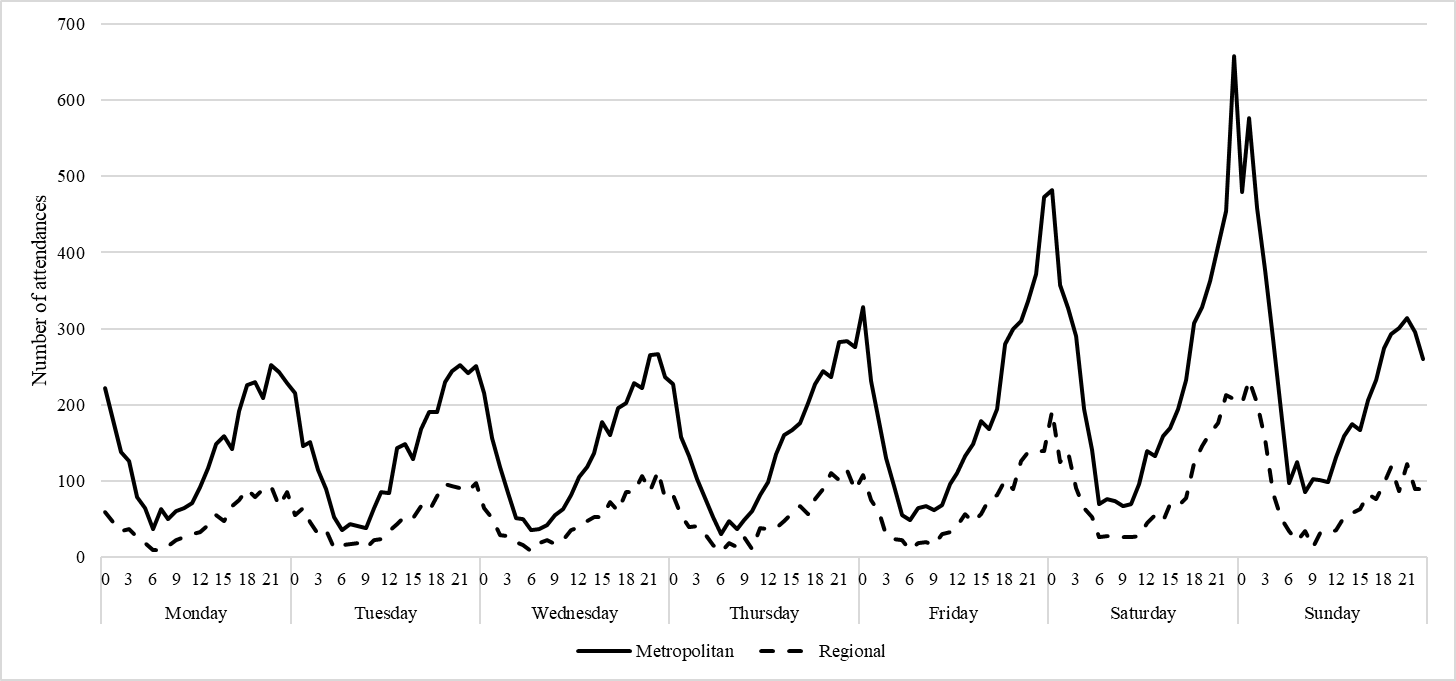


Figure S2. 2019 alcohol-involved ambulance attendances by regionality, Victoria, Australia


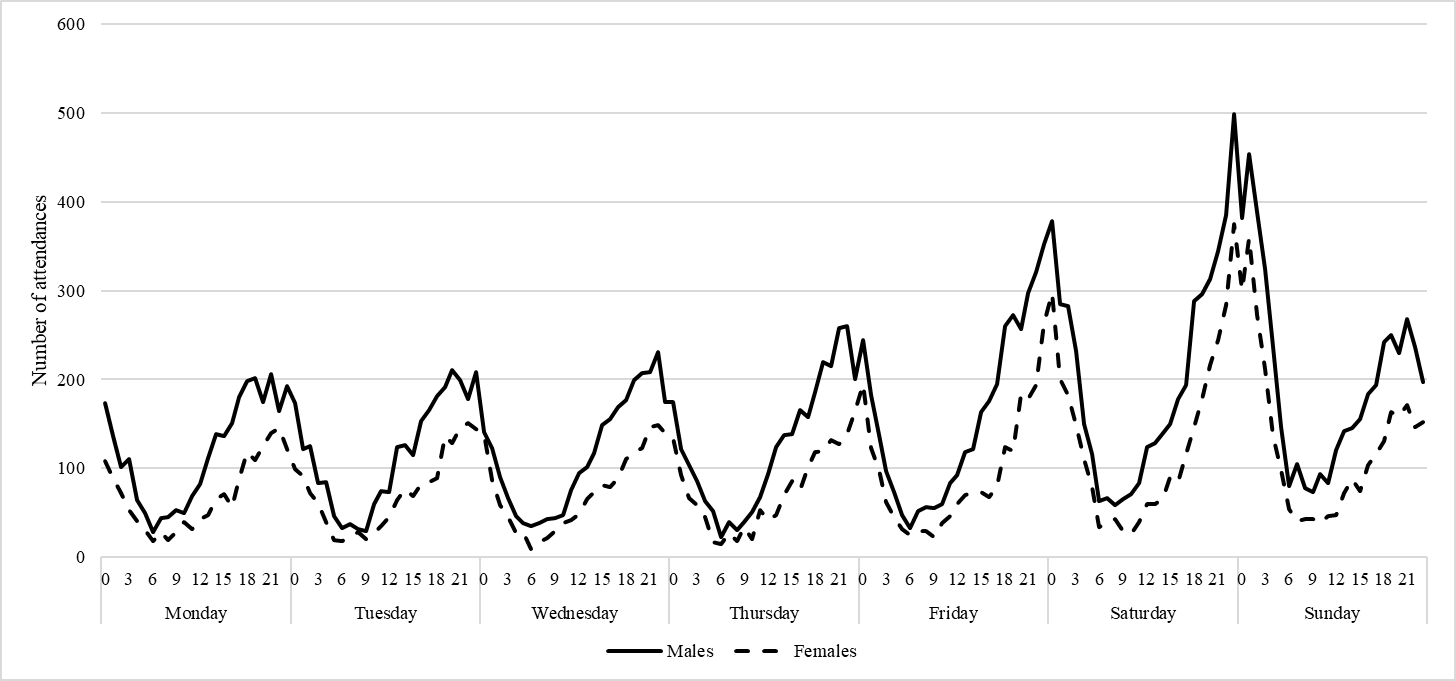


Figure S3. 2019 alcohol-involved ambulance attendances by gender, Victoria, Australia


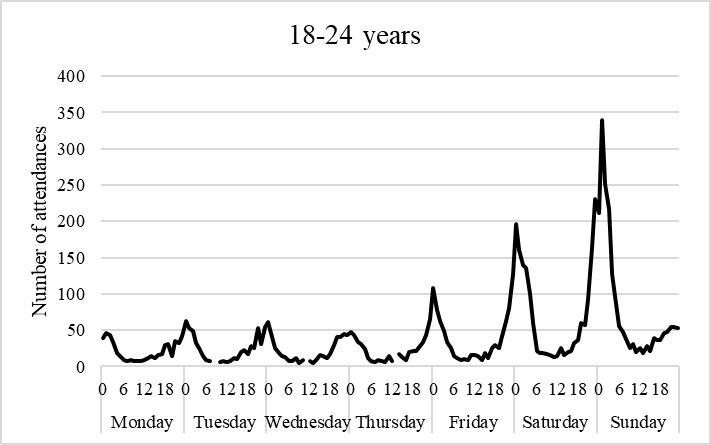

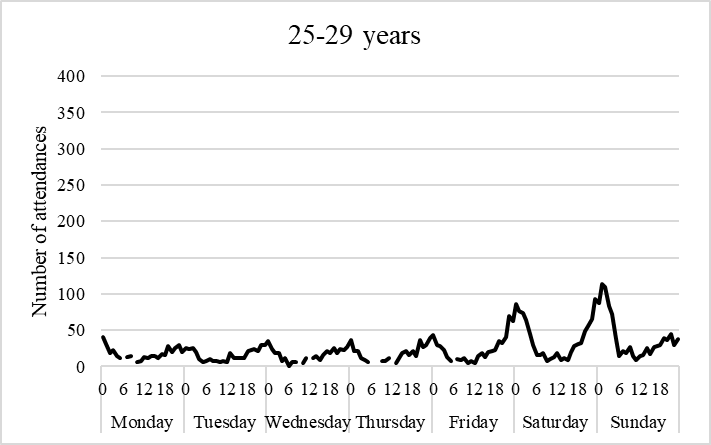

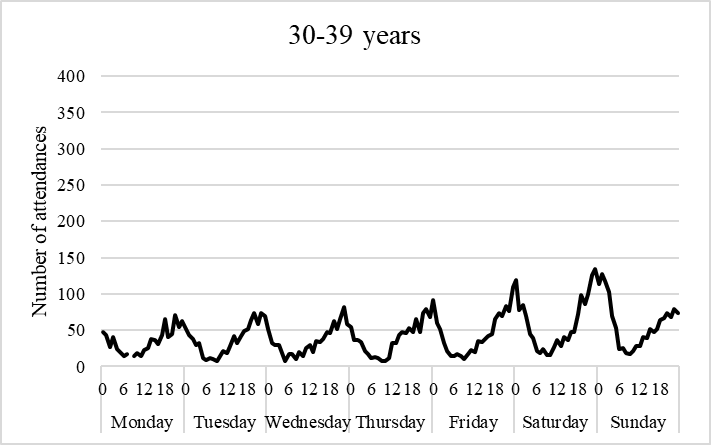

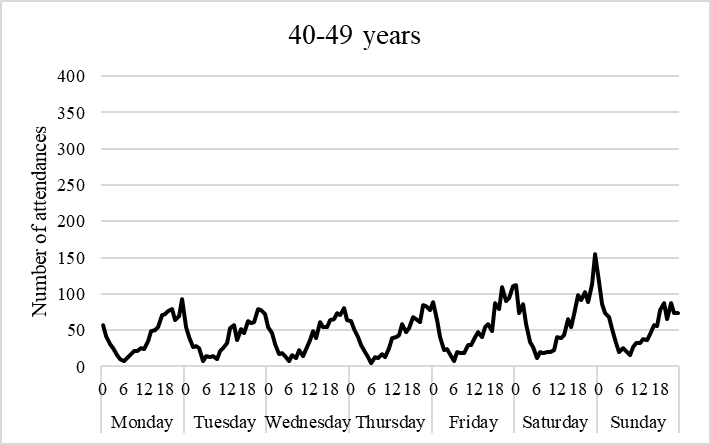

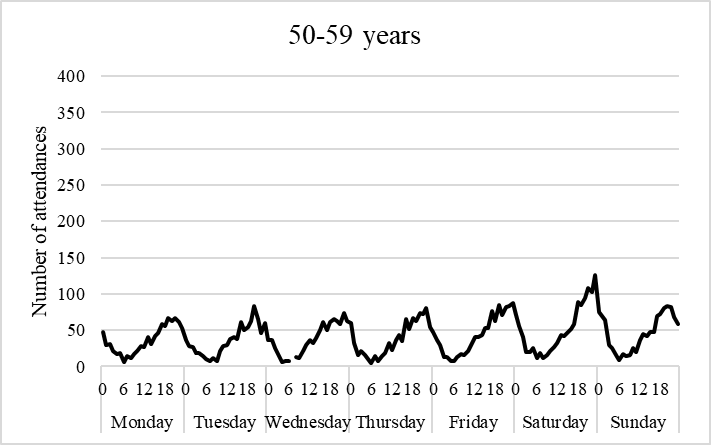

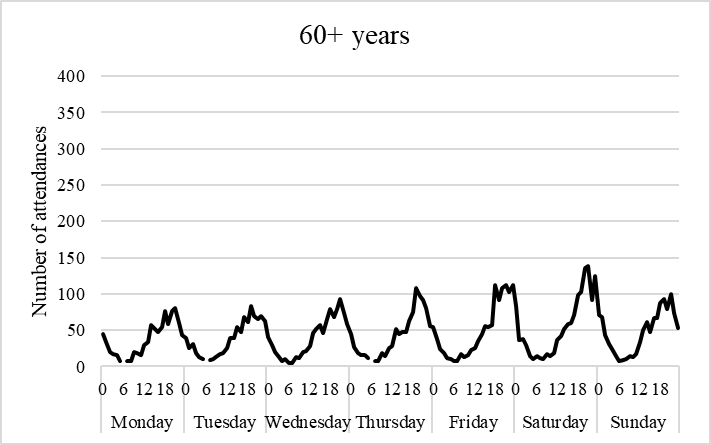


Figure S4. 2019 alcohol-involved ambulance attendances by age group, Victoria, Australia

*Note. Gaps are due a cell size of fewer than 5 attendances*


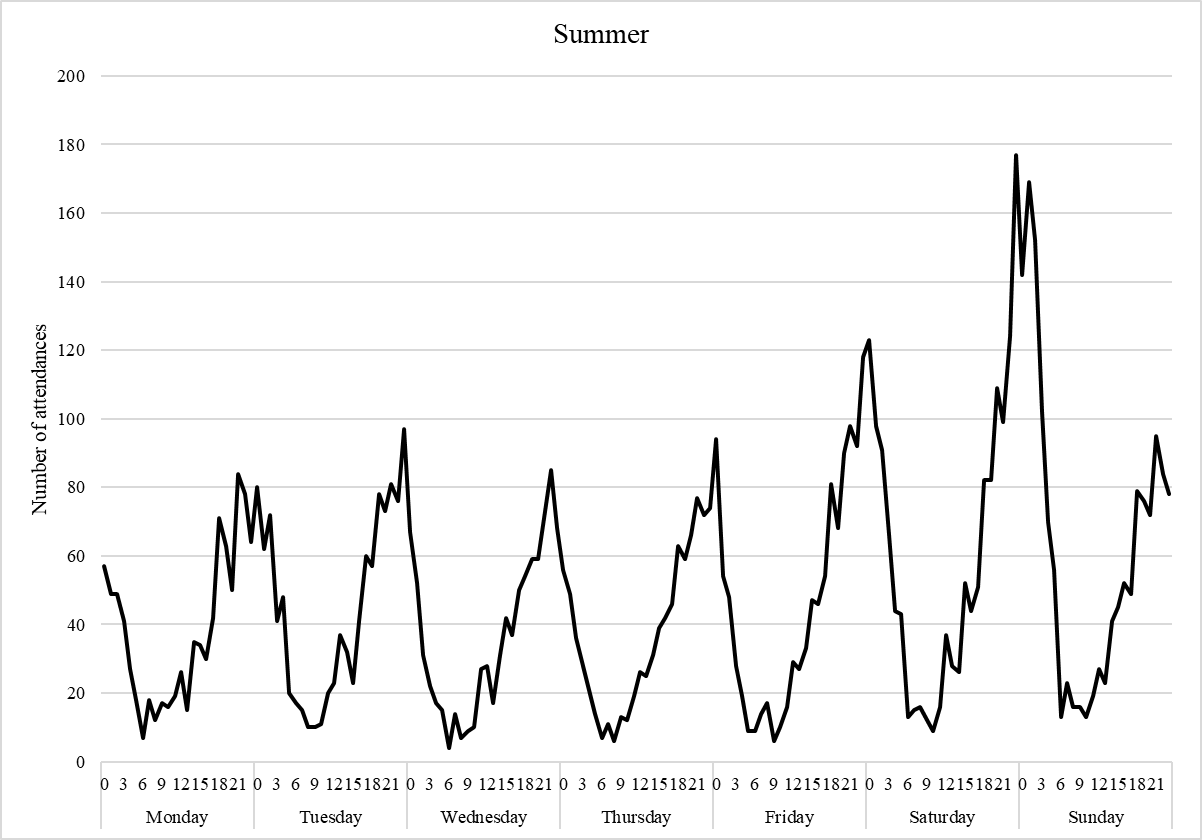

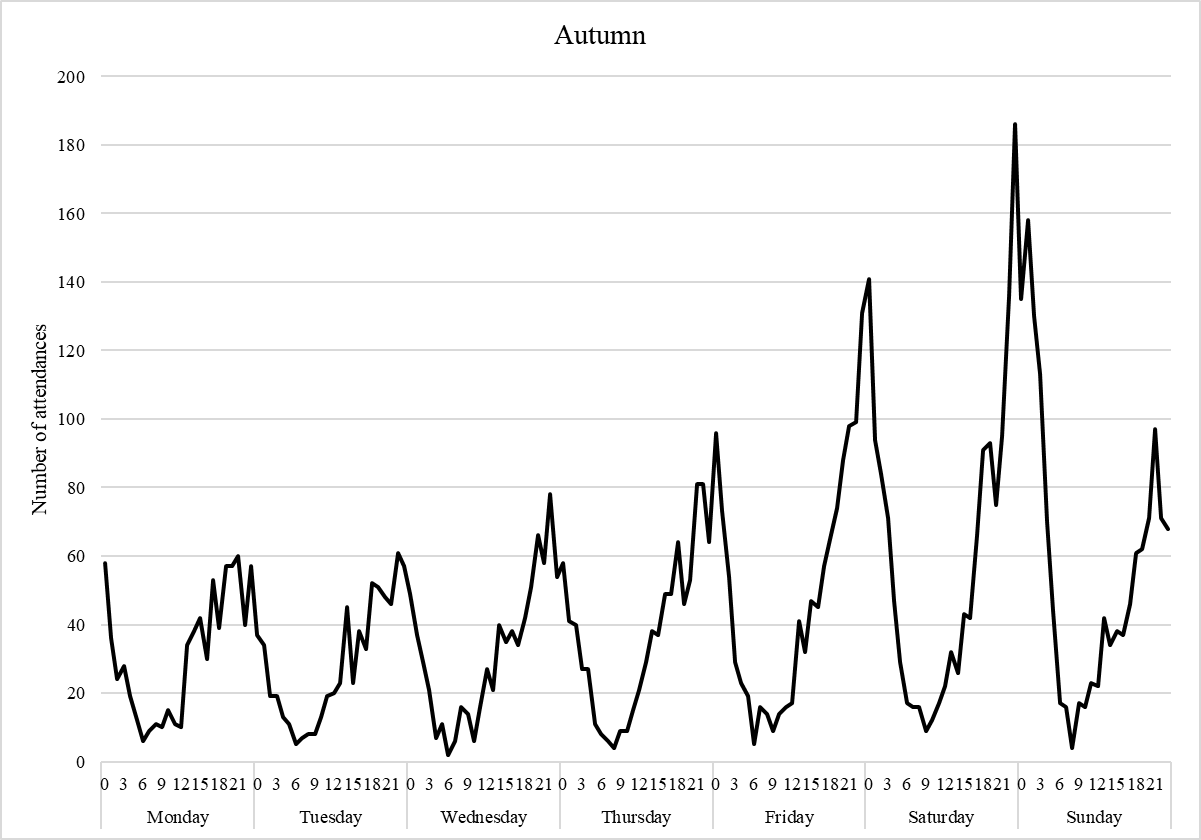


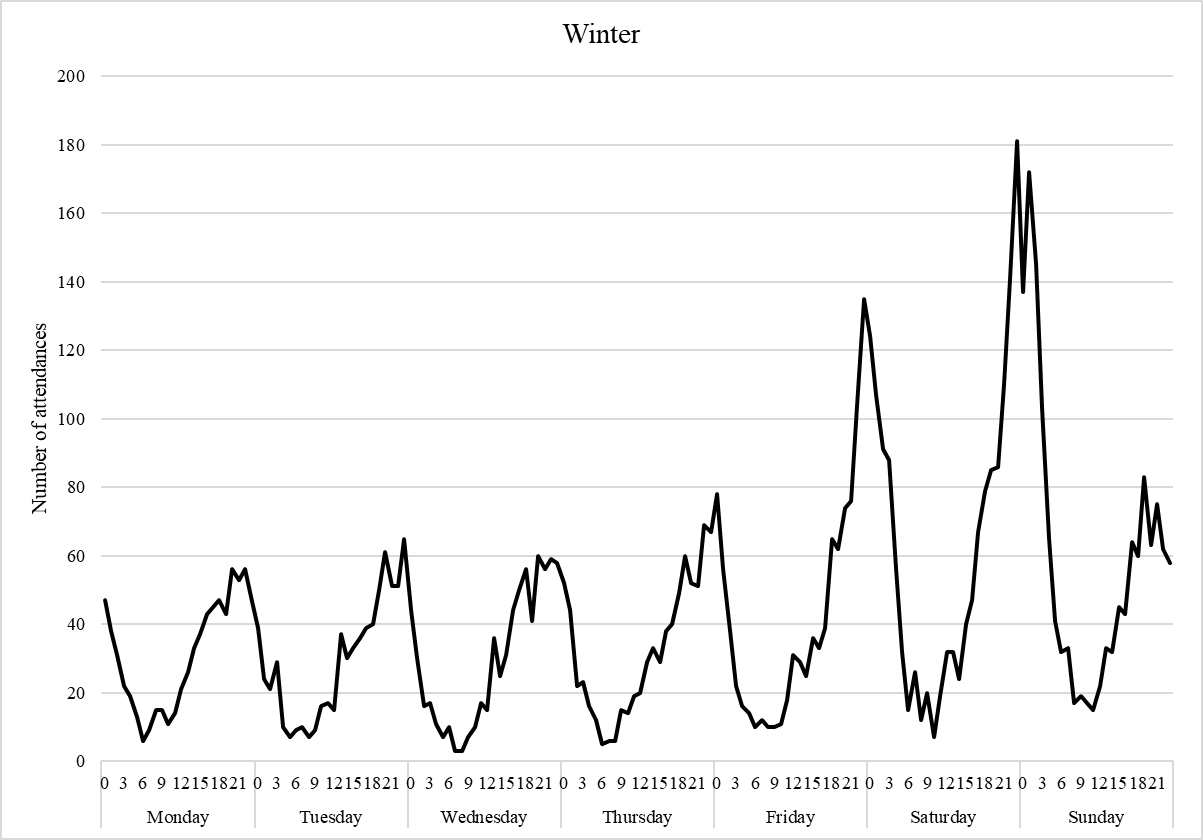

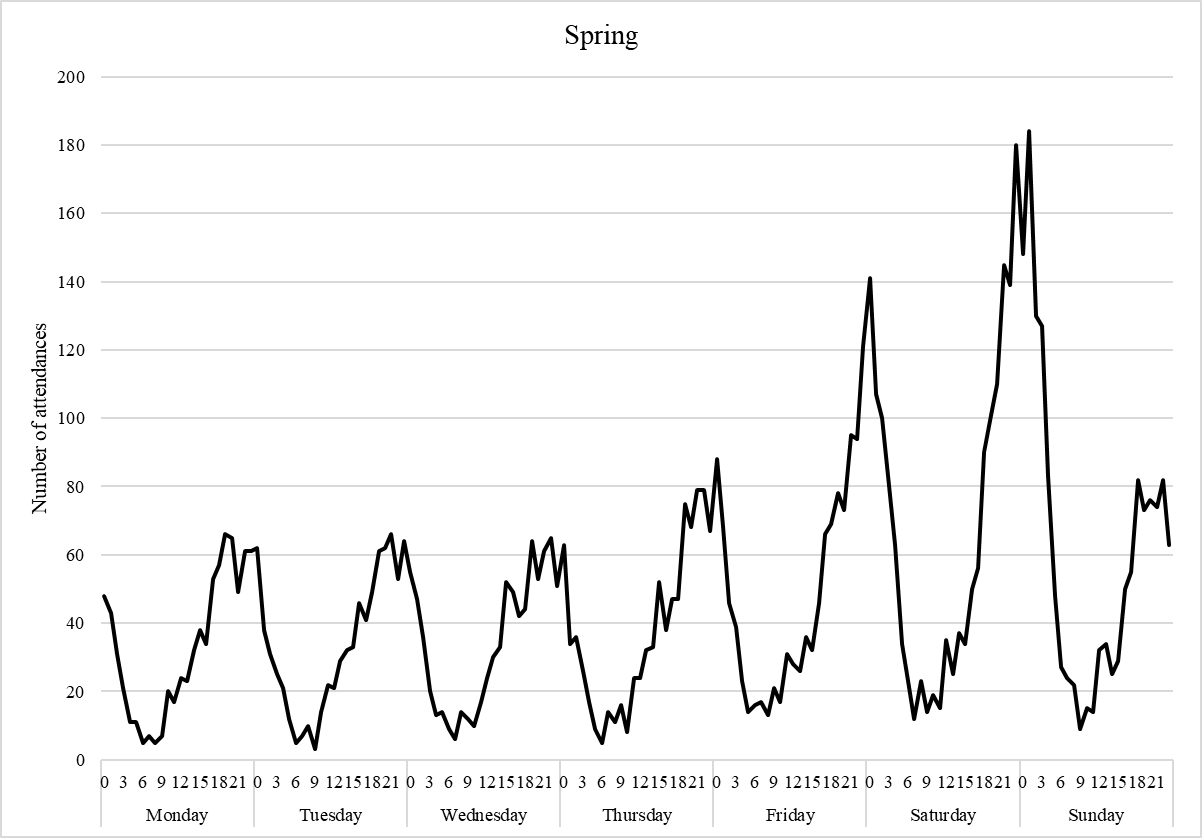


Figure S5. 2019 alcohol intoxication ambulance attendances by season, Victoria, Australia


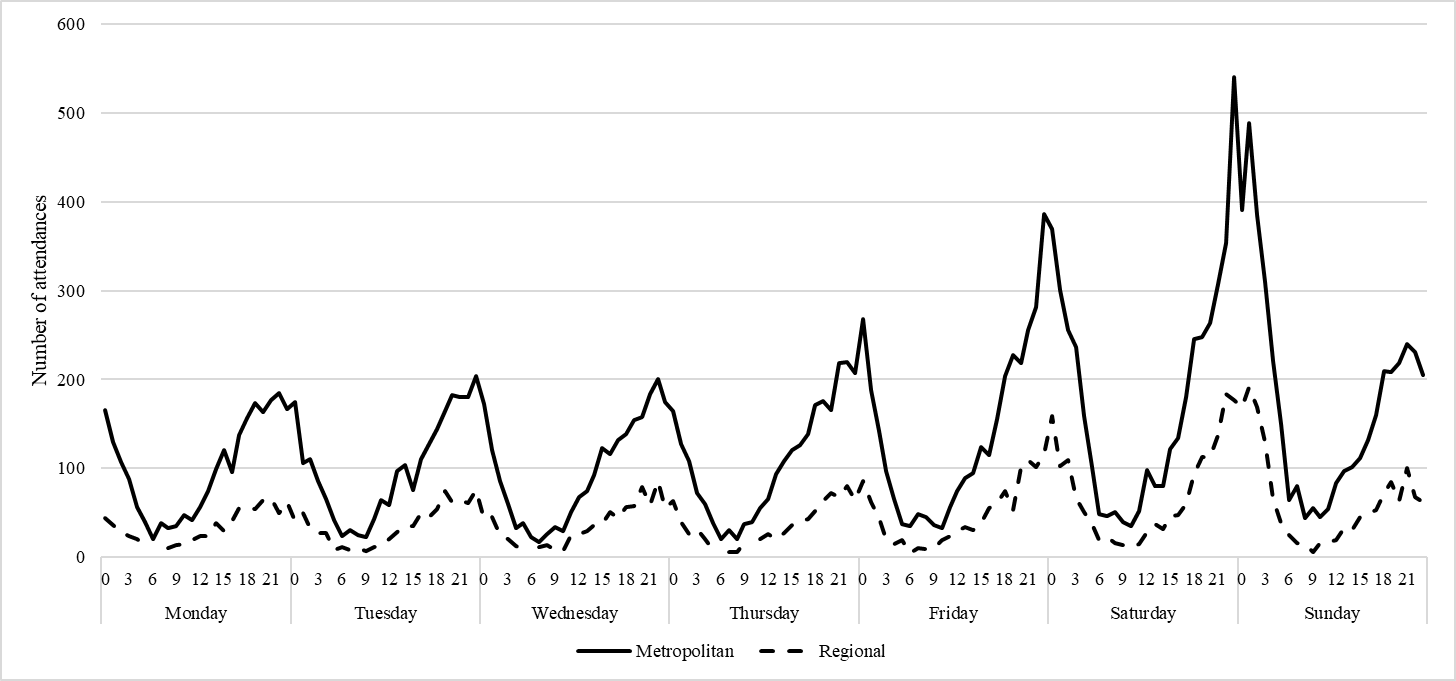


Figure S6. 2019 alcohol intoxication ambulance attendances by regionality, Victoria, Australia

*Note. Gaps are due a cell size of fewer than 5 attendances*


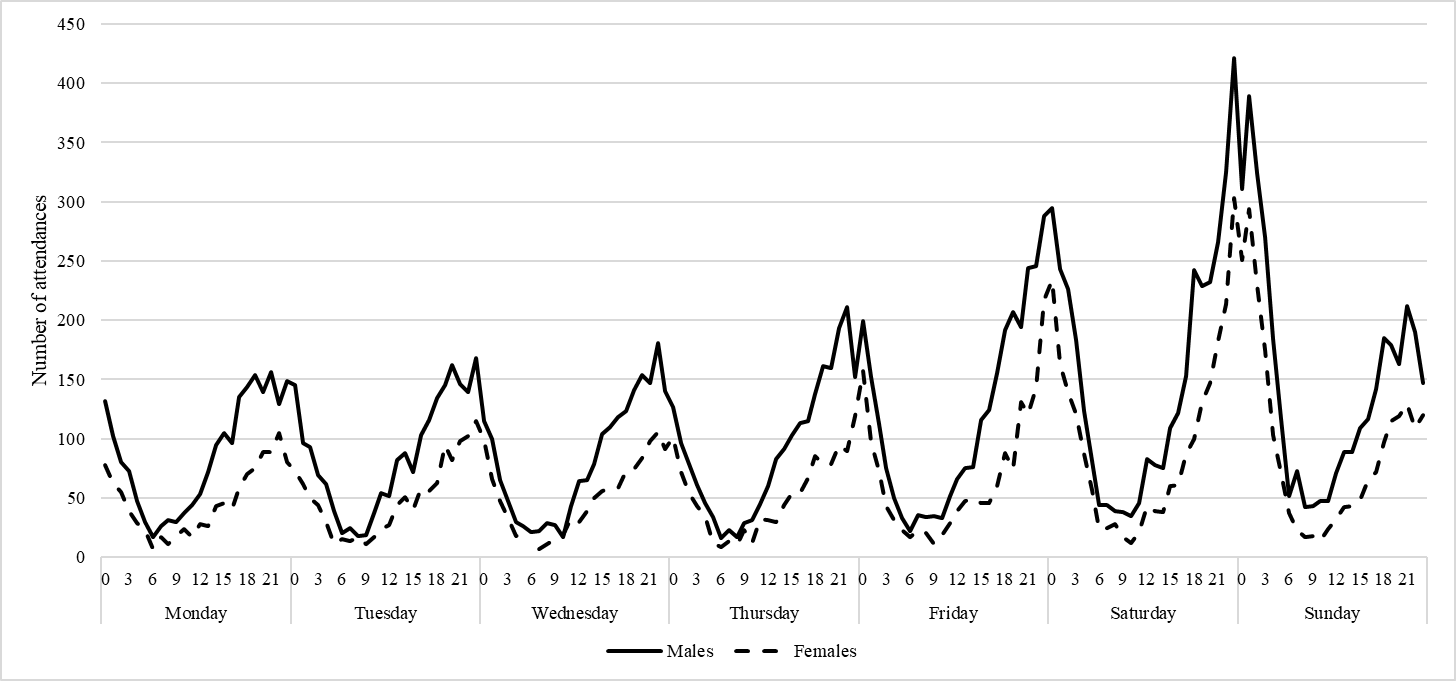


Figure S7. 2019 alcohol intoxication ambulance attendances by gender, Victoria, Australia

*Note. Gaps are due a cell size of fewer than 5 attendances*


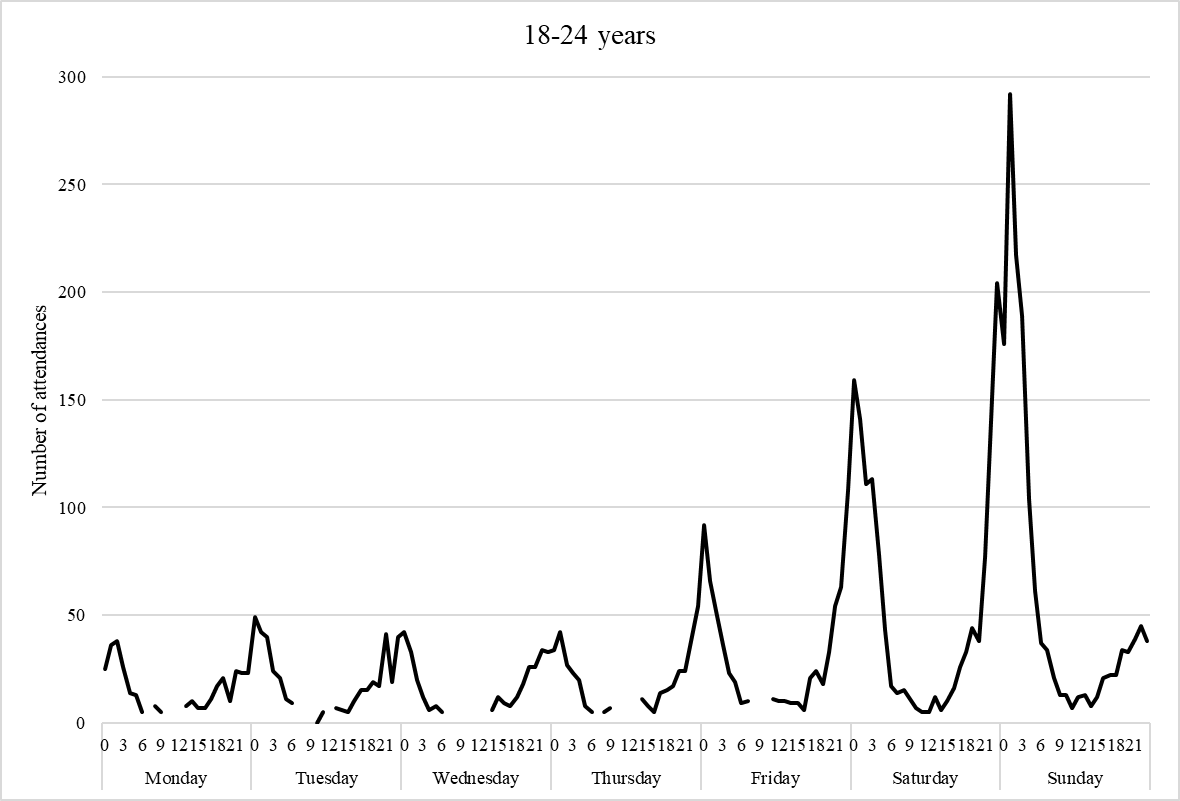

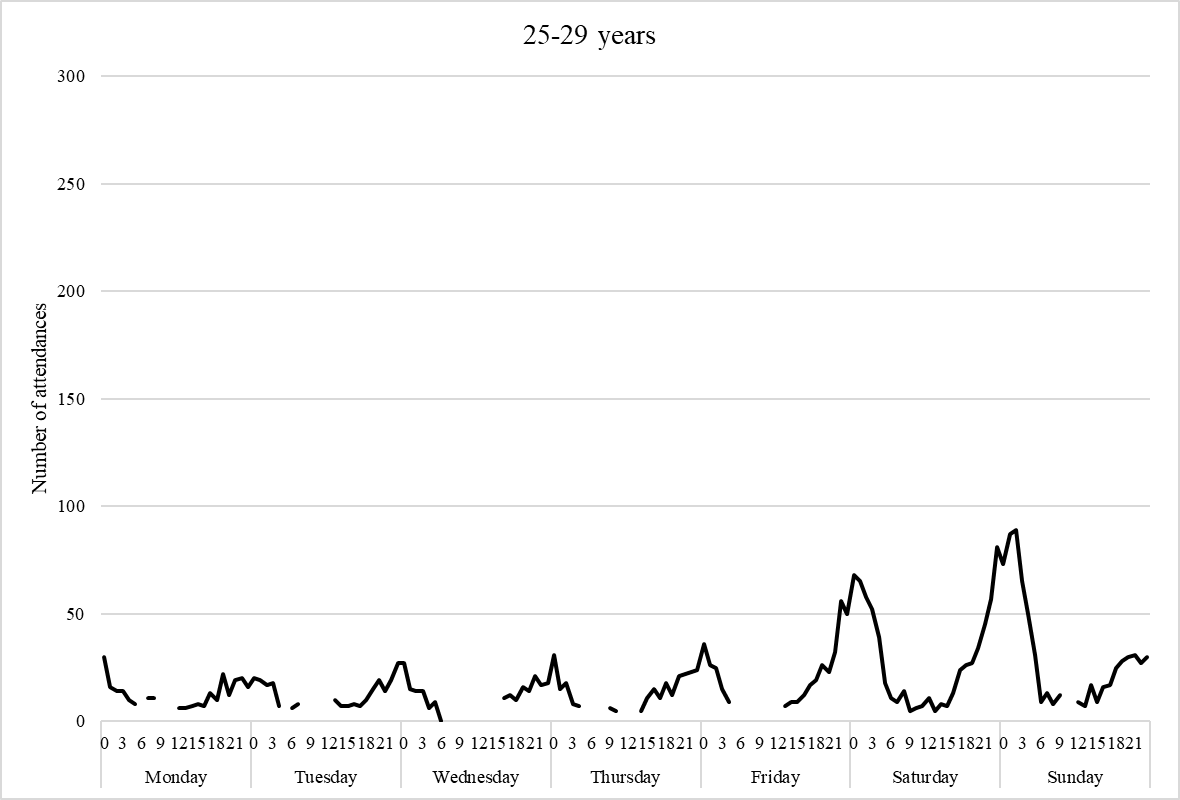

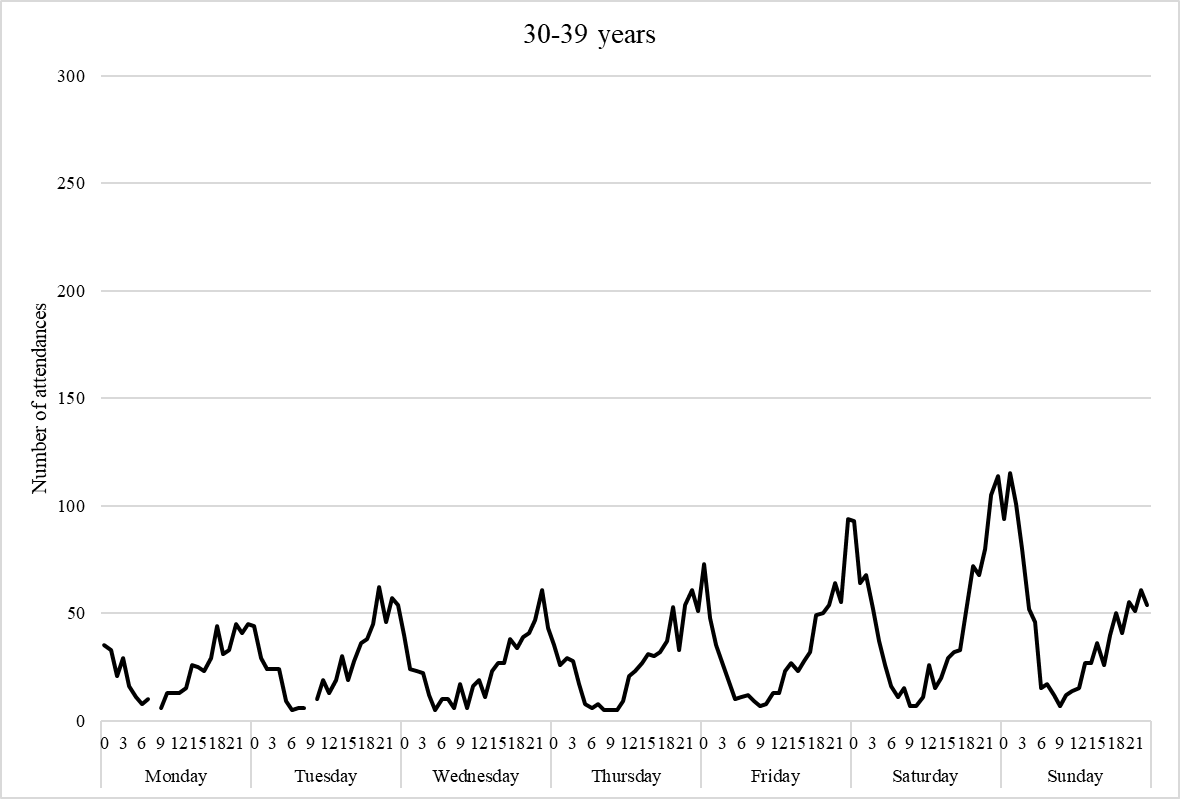

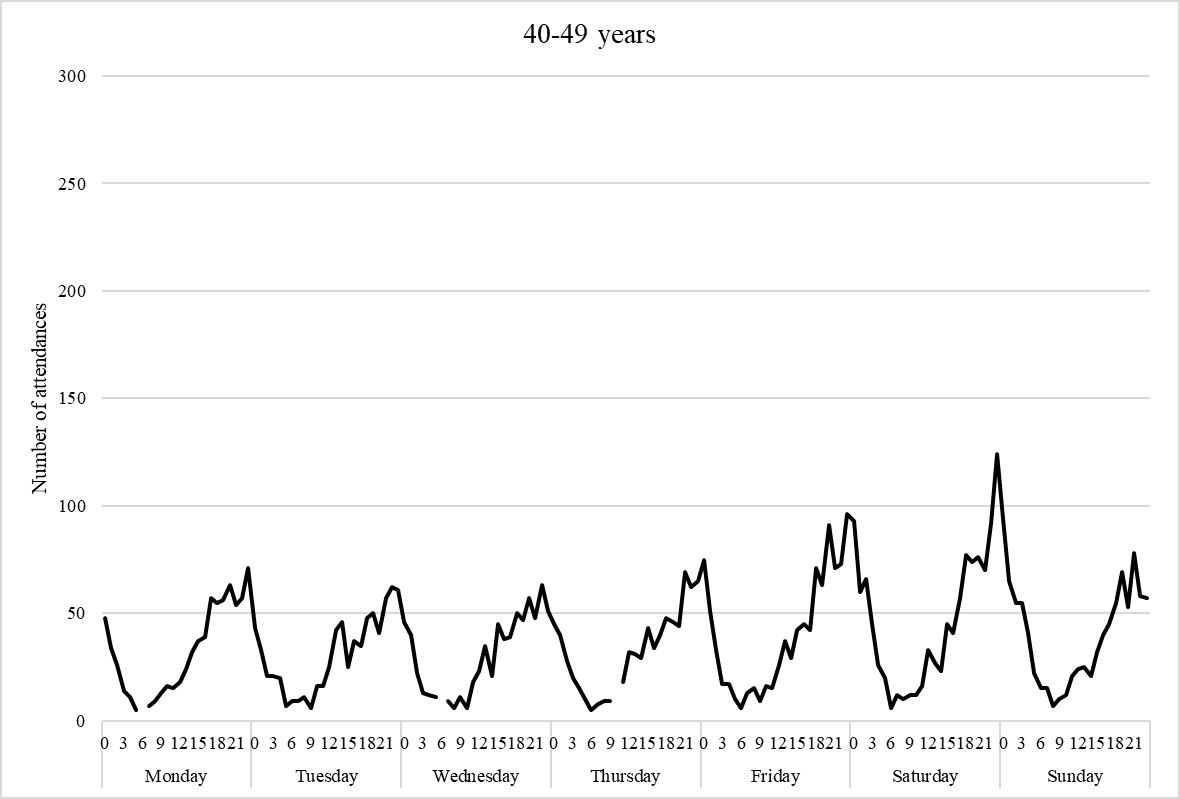


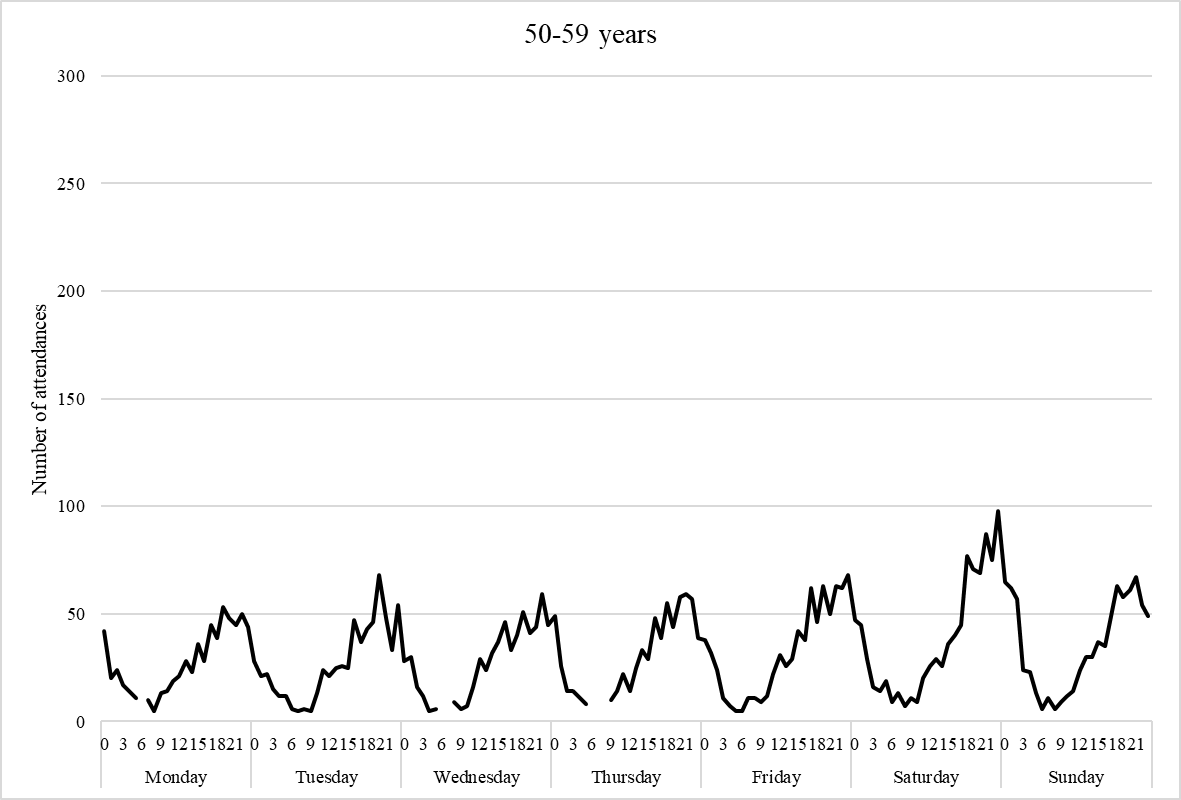

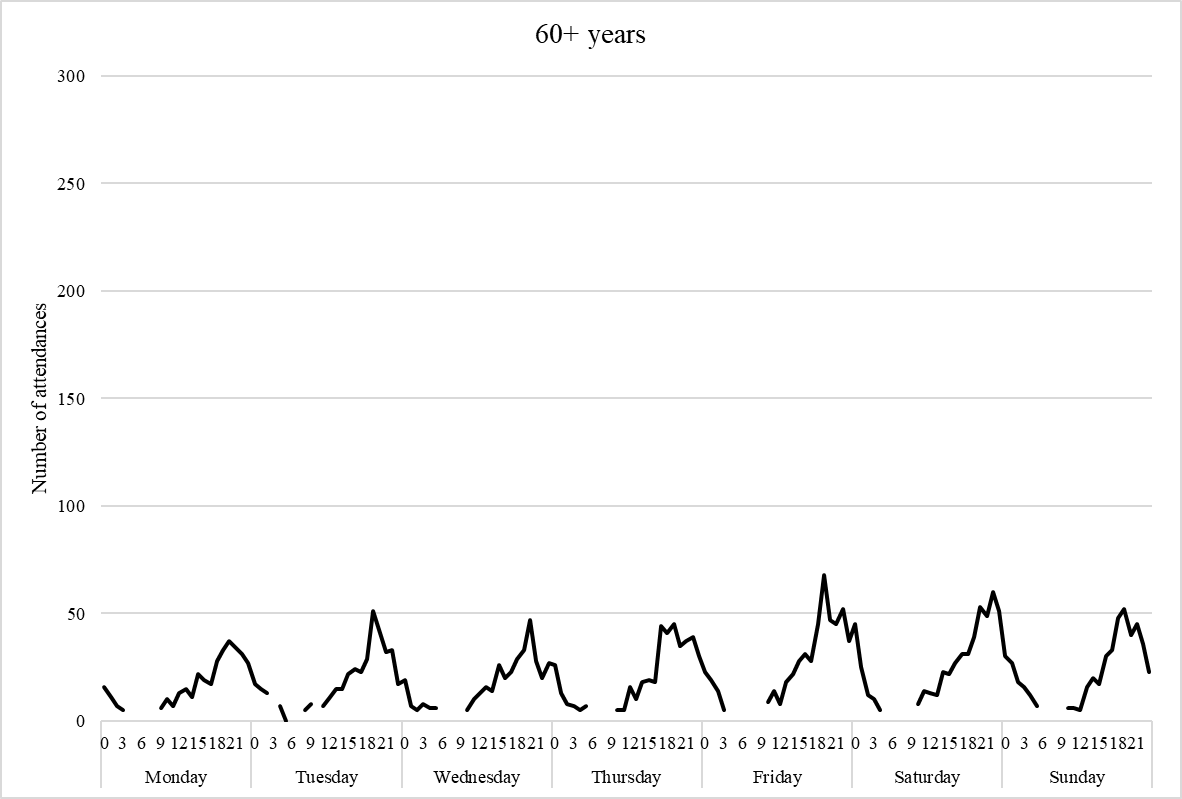


Figure S8. 2019 alcohol intoxication ambulance attendances by age group, Victoria, Australia

*Note. Gaps are due a cell size of fewer than 5 attendances*
