# Supplementary material for: Description of Trends over the Week in Alcohol-Related Ambulance Attendance Data
Source: Int J Environ Res Public Health. 2023 Apr 19;20(8):5583. doi: 10.3390/ijerph20085583 (PMC10138978; doi:10.3390/ijerph20085583)
Supplement: Supplementary file 1 [file ijerph-20-05583-s001.zip › Ambo_HAH_Tables S1 & S2.docx]

**Table S1.** Moderate temporal peaks for Victorian alcohol-involved ambulance attendance data, 2019

| **Season** | **Summer** | | **Autumn** | | **Winter** | | **Spring** | |
| --- | --- | --- | --- | --- | --- | --- | --- | --- |
|  | Tuesday 7pm to 11:59pm  Thursday 8pm to Friday 0:59am  Sunday 6pm to 11:59pm | | Thursday 8pm to Friday 1:59am  Sunday 6pm to 11:59pm | | Thursday 7pm to Friday 0:59am  Sunday 5pm to 10:59pm | | Thursday 7pm to Friday 0:59am  Sunday 6pm to 11:59pm | |
| **Region** | **Metropolitan** | | | | **Regional** | | | |
|  | Thursday 7pm to Friday 0:59am  Sunday 6pm to 11:59pm | | | | Thursday 7pm to Friday 0:59am  Sunday 6pm to 11:59pm | | | |
| **Gender** | **Males** | | | | **Females** | | | |
|  | Thursday 7pm to Friday 0:59am  Sunday 6pm to 11:59pm | | | | Thursday 8pm to Friday 0:59am  Sunday 6pm to 11:59pm | | | |
| **Age** | **18-24 years** | **25-29 years** | | **30-39 years** | **40-49 years** | **50-59 years** | | **60+ years** |
|  | Sunday 7pm to 11:59am | Thursday 8pm to Friday 1:59am  Sunday 6pm to Monday 1:59am | | Thursday 9pm to Friday 0:59am  Sunday 6pm to 11:59pm | Thursday 9pm to Friday 0:59am  Sunday 6pm to 11:59pm | Monday 7pm to 10:59pm  Thursday 4pm to 10:59pm  Sunday 5pm to 10:59pm | | Wednesday 5pm to 10:59pm  Thursday 6pm to 10:59pm  Sunday 5pm to 10:59pm |
| **Victoria wide** | Thursday 7pm to Friday 0:59am  Sunday 6pm to 11:59pm | | | | | | | |

**Table S2.** Moderate temporal peaks for Victorian alcohol intoxication ambulance attendance data, 2019

| **Season** | **Summer** | | **Autumn** | | **Winter** | | **Spring** | |
| --- | --- | --- | --- | --- | --- | --- | --- | --- |
|  | Tuesday 7pm to 11:59pm  Thursday 9pm to Friday 0:59am  Sunday 6pm to 11:59pm | | Thursday 9pm to Friday 1:59am  Sunday 6pm to Monday 0:59pm | | Sunday 5pm to 11:59pm | | Thursday 7pm to Friday 1:59am  Sunday 6pm to 11:59pm | |
| **Region** | **Metropolitan** | | | | **Regional** | | | |
|  | Thursday 9pm to Friday 1:59am  Sunday 6pm to 11:59pm | | | | Thursday 7pm to Friday 1:59am  Sunday 6pm to 11:59pm | | | |
| **Gender** | **Males** | | | | **Females** | | | |
|  | Thursday 7pm to Friday 1:59am  Sunday 6pm to 10:59pm | | | | Thursday 9pm to Friday 0:59am  Sunday 6pm to 11:59pm | | | |
| **Age** | **18-24 years** | **25-29 years** | | **30-39 years** | **40-49 years** | **50-59 years** | | **60+ years** |
|  | Thursday 10pm to Friday 2:59am | Thursday 11pm to Friday 2:59am  Sunday 6pm to Monday 0:59am | | Tuesday 7pm to 11:59pm  Thursday 7pm to Friday 1:59am  Sunday 6pm to 11:59pm | Monday 5pm to 11:59pm  Thursday 9pm to Friday 0:59am  Sunday 6pm to 11:59pm | Thursday 6pm to 10:59pm  Sunday 5pm to 11:59pm | | Thursday 5pm to 10:59pm  Sunday 5pm to 10:59pm |
| **Victoria wide** | Thursday 7pm to Friday 1:59am  Sunday 6pm to 11:59pm | | | | | | | |
